# Targeting the CCL2-CCR2 axis in depressive disorders

**DOI:** 10.1007/s43440-021-00280-w

**Published:** 2021-05-24

**Authors:** Katarzyna Curzytek, Monika Leśkiewicz

**Affiliations:** Department of Experimental Neuroendocrinology, Laboratory of Immunoendocrinology, Maj Institute of Pharmacology, Polish Academy of Sciences, 12 Smętna St., 31-343 Kraków, Poland

**Keywords:** CCL2, CCR2, Depression, Antidepressant drugs, Affective disorders

## Abstract

Since affective disorders are considered to be underlain by the immune system malfunction, an important role in their pathophysiology is assigned to the proinflammatory mediators. Recently, chemokines, the group of chemotactic cytokines, have become a focus for basic and clinical scientists in the context of the development and treatment of brain diseases. Among them, chemokine CCL2 and its main receptor CCR2 have become candidate mediators of abnormal brain-immune system dialogue in depression. Besides the chemotactic activity, the CCL2-CCR2 axis is involved in various neurobiological processes, neurogenesis, neurotransmission, neuroinflammation, neurodegeneration, as well as neuroregeneration. Given the range of immunomodulatory possibilities that the CCL2-CCR2 pair can exert on the nervous system, its proinflammatory properties were initially thought to be a major contributor to the development of depressive disorders. However, further research suggests that the malfunctions of the nervous system are rather associated with impaired homeostatic properties manifested by the CCL2-CCR2 dyad dysfunctions. This review aims to present literature data on the action of the CCL2-CCR2 axis in the central nervous system under physiological and pathological conditions, as well as the contribution of this ligand-receptor system to the processes underlying affective disorders. Additionally, this article draws attention to the importance of the CCL2-CRR2 pathway as a potential pharmacological target with antidepressant potential.

## Introduction

In light of the recent SARS-CoV-2 coronavirus pandemic, the mechanisms underlying the immune system regulation have become the subject of extensive studies. The important role of the immunological component in these diseases has been proposed much earlier by the scientists involved in research on the pathophysiology and treatment of psychiatric disorders. Depression was the first mental illness found to be significantly associated with the immune system disorders. Initially, proinflammatory cytokines were considered as the main player in the emergence of depression, however, recently the focus has been shifted to chemokines and their receptors, the homeostasis of which is disturbed during affective disorders. One of them, chemokine C–C motif ligand-2 (CCL2, known also as monocyte chemoattractant protein 1, MCP-1) and its major receptor (CCR2) have been shown to act as mediators of neuroinflammation, neurogenesis, and synaptic transmission, along with plasticity [1–3]. The wide expression of this chemokine and CCR2 has been demonstrated under basal as well as pro-inflammatory conditions on multiple types of cells within the central nervous system [2, 4]. Therefore, CCL2 became an essential candidate linking peripheral and central inflammation and mediating the neuroimmune crosstalk, due to its role in cellular migration and immune coordination.

## Biology of CCL2 and its receptors

CCL2 is located on chromosome 17 (chr.17, q11.2). The human version of this chemokine is composed of 76 amino acids with a molecular weight of 13 kDa [5]. CCL2 belongs to the CC chemokine family. Based on the location of the key cysteine residues (N-terminal region of the protein) that participate in disulphide bonding, CCL2 was assigned to the CC chemokine family.[6].

**Fig. 1:**
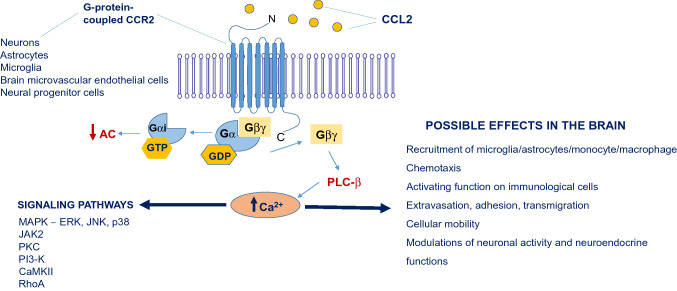
CCL2-CCR2 interaction and signaling molecules participating in the regulation of the inflammatory activation state of the brain cells. *AC* adenylate cyclase, *PLC-β* phospholipase c-β, *PKC* protein kinase C, *MAPK* mitogen-activated protein kinases, *ERK* extracellular signal-regulated kinase, *JAK2* Janus kinase 2, *JNK* c-jun N-terminal kinase, *PI3-K* phosphoinositide 3-kinase, *CaMKII* Ca^2+^/calmodulin-dependent protein kinase, *RhoA* cytoplasmatic protein belonging to the family of small GTPases

CCL2 was the first identified human CC chemokine and plays a key role in the regulation of migration and infiltration of monocytes/macrophages, memory T cells, and NK cells. Apart from its chemotactic properties, this chemokine increases the adhesion of monocytes and influences the polarization of T lymphocytes towards the Th2 type [7]. CCL2 is produced mainly by monocytes/macrophages, however, other cell types, including endothelial, fibroblast, epithelial, smooth muscle, mesangial, neuronal, astrocytic, and microglial cells also release this chemokine [8]. CCL2 can form homodimers, heterodimers, and even higher-order aggregates [9] (Fig. 1).

The binding of chemokines to their cognate receptors triggers signal transduction pathways and, hence, biological effects, such as chemotaxis. These receptors belong to the superfamily of seven transmembrane-domain G-protein-coupled receptors (GPCRs). GPCRs are integral membrane proteins composed of a short N-terminal extracellular domain, seven hydrophobic conserved transmembrane domains linked by three intracellular and extracellular loops, and a serine/threonine-rich C-terminal intracellular region, the latter of which is coupled to a heterotrimeric G-protein affecting intracellular signaling [10] (Fig. 1). Like many other chemokines, CCL2 can bind to multiple receptors. It interacts with different GPCRs: CCR1, CCR2, CCR4, CCR10, but also two atypical receptors: ACKR1 and ACKR2 (non-G protein-coupled receptors) [5, 9–11]. Preferentially, CCL2 binds to CCR2, which is present in various peripheral tissues, including blood, heart, kidney, liver, lung, ovary, pancreas, spinal cord, spleen, and thymus, and also in the brain [10]. It should be noted that similarly to its ligand, CCR2 can cooperate with several proteins. For example, it can be activated by CCL7, CCL8, and CCL13 or inhibited by CCL11 and CCL26. This receptor can also interact with other chemokine receptors, like CCR5 or CXCR4 [9]. CCR2 appears in two isoforms: CCR2A and CCR2B, which result from alternative splicing, yet, differ only in their C-terminal tails [12]. CCR2B is the dominant isoform and accounts for 90% of all CCR2 expression, for instance, on monocytes, NK cells, as well as microglia, astrocytes, and neurons, while CCR2A production is limited to a small subset of mononuclear and smooth muscle cells [5, 7, 12, 13]. Furthermore, Hughes & Nibbs' “Guide to chemokines and their receptors” [9] shows the complexity of the posttranslational processes that chemokines undergo, e.g., CCL2 nitration may impair chemotactic capacity via CCR2.

A variety of signal transduction pathways is activated by chemokine receptors, leading to either diverse cellular responses, depending on the specific receptor-chemokine pair, or similar effects. The dual biological outcome indicates a multitude of functions performed by chemokines. These mechanisms can alter gene expression and downstream changes in protein production. Therefore, regulation of protein levels could be the proposed mechanism through which CCL2 participates in homeostatic control of neuronal and glial functions.

## CCL2—possible effects in the brain

Currently, it has been postulated that chemokines and their receptors play a pivotal role in the central nervous system (CNS) for microglia, astrocytes, neurons, and neural stem cells as well as infiltrating immune cells during neuroinflammation [8]. Chemokines are involved in the intercellular communication, proliferation, differentiation, and survival of various types of neurons [14]. Their activity is especially relevant in the cerebral cortex, hippocampus, and hypothalamus, structures involved in the pathogenesis of affective disorders [15].

### CCL2 and brain development, neuronal plasticity, and neurotransmission

The significant effect of CCL2 on the nervous system has been observed already at the early stage of brain development. Treatment of rat embryonic cells with CCL2 and CCL7 enhanced the differentiation of neurons towards the dopaminergic phenotype [16]. At the later developmental stages, an upregulated level of CCL2 was correlated with the increase in the excitability of dopaminergic neurons and the intensification of locomotor activity in rats [17]. Further evidence came from research conducted among 5-year-old children that showed the elevation of CCL2 production in the serum, correlated with a greater probability of attention-deficit hyperactivity disorder (ADHD) occurrence [18]. Additionally, anatomical studies carried out on human brains revealed that CCL2 might be associated with the development and maturation of Purkinje cells, dentate nucleus, the inferior olivary nucleus, and their network, and can promote the growth of dendrites and synapses. Contrary, CCL2-elevated levels were observed during abnormal neuronal development and ischemia damage [19]. Complementary data, obtained from studies conducted in transgenic mice, revealed that chronic exposure of hippocampal slices to higher levels of CCL2 in the brain (particularly in the hippocampus) resulted in the reduced synaptic transmission and neuronal excitability, as well as enhanced short-term synaptic plasticity with functional consequences on the brain connectivity, learning, memory and the behavioral performance [20]. The other electrophysiological studies demonstrated that the application of CCL2 onto different areas of hippocampal neurons during the early phase of systemic inflammation enhanced excitatory synaptic transmission and total neuronal excitability in normal mice but not in *Ccr2* knockout mice [21]. A report by Zhou et al. [22] also confirmed the impact of CCL2 on neuronal excitability and synaptic transmission. Moreover, electrical recordings in neurons of isolated spinal cord slices showed that treatment with CCL2 enhanced spontaneous EPSCs and potentiated NMDA- and AMPA-induced currents [23]. Using the patch-clamp method in a similar model, it was also proved that CCL2 inhibited GABA(A)-mediated GABAergic responses [24].

Further support for the important role of CCL2 in the pathogenesis of depression came from the observation that this chemokine is involved in the release of certain neurotransmitters in the brain. CCL2 is one of the chemokines affecting synaptic responses, neuronal activity, or neuronal ion channel function (reviewed in [14]). For example, CCL2 co-localizes with acetylcholine in the substantia innominate and the oculomotor nucleus, and with dopamine in the substantia nigra [17, 25].

These data support the concept that chemokines act as neuromodulators in the CNS, serving as critical signaling molecules for neural-glial interaction.

### CCL2-CCR2 and neuroendocrine function

The immunohistochemical studies revealed co-localization of CCL2 with melanin-concentrating hormone-expressing (MCH) neurons and vasopressin in magnocellular neuronal cell bodies [26]. Some behavioral experiments demonstrated that CCL2 possesses stress-related neuroendocrine functions. It was discovered that exposure to restraint stress in rats caused a moderate increase in CCL2 because of the inhibitory action of glucocorticoids secreted during a stress response. Additionally, the use of metyrapone, a selective glucocorticoid synthesis inhibitor, during the stress procedure, caused a large increase in CCL2 production in the cortex of the tested animals [27]. There is also evidence that CCL2 controls feeding behavior and body weight loss by influencing CCR2 in the hypothalamic orexigenic neuropeptide neurons. Interestingly, this effect was completely abolished in mice with *Ccr2* knockout or treated with the CCR2 antagonist [28]. These data suggest that CCL2 could act also as a modulator of neuroendocrine functions.

### CCL2-CCR2 in the response of the immune cells in the brain

CCL2 is considered as a proinflammatory chemokine that regulates the chemotaxis of monocyte-derived macrophages, T lymphocytes, and dendritic cells to mediate neuroinflammation in the CNS [29]. It has been shown that the CCL2-CCR2 axis can activate microglia and influence the secretion of proinflammatory factors such as interleukin-1β (IL-1β) and IL-18 [2]. On the other hand, these interleukins can enhance the response of the CCL2-CCR2 dyad to their action through a feedback mechanism.[30]. Studies carried out in our laboratory have shown that microglial cells isolated from prenatally stressed animals produced higher levels of IL-1β and IL-18. Moreover, microglial cells obtained from those rats were characterized by increased expression of CCL2 and CCR2 [31]. Our subsequent research using the same experimental conditions confirmed similar observations in the entire structures of the brain, i.e., the hippocampus and frontal cortex [3]. Although the role of CCL2 in the activation of microglia and astrocytes in neuroinflammation is well documented [2, 31, 32], recent studies have shown that PDGFRβ cells (mural cells of blood vessels) are the significant source of CCL2 during early neuroinflammation [21]. Robust experimental evidence indicates that elevated CCL2 levels induce the recruitment of macrophages, production of cytokines, and direct alteration of the expression of endothelial cell tight-junction proteins to increase blood–brain barrier (BBB) permeability, observed during various pathological processes, such as multiple sclerosis [33, 34], stroke [35, 36], and Alzheimer’s disease [37, 38].

CCL2 intensifies monocyte adhesion and influences the polarization of T lymphocytes towards Th2, which enhances the expression of anti-inflammatory cytokines, e.g., IL-4 [39]. Besides, macrophage influx in response to an increase in CCL2 may enhance post-stroke regeneration via phagocytosis of myelin debris. Furthermore, CCL2 secreted by activated glial cells attracts neural precursor cells and thus, may influence repair after injury by amplifying neurogenesis [4]. Other studies also disclosed the functional benefits of the CCR2 over-expression, specifically alleviating post-stroke cognitive impairment by enhancing microglia/macrophage M2 polarization and probably through suppressing the CCL2-induced hematogenous macrophage migration and activation [40].

### Neuroprotective effects of CCL2 in the brain cells

Although the elevated CCL2 is mainly associated with the pathophysiology of numerous diseases, its pleiotropic activities are related to beneficial effects. The results of some studies indicate that CCL2 may function as a protective protein in the CNS by inhibition of necrotic and apoptotic processes in different models of cell damage.

In vitro studies provided evidence on the neuroprotective activity of CCL2 against NMDA toxicity in mixed cortical cultures [41]. Moreover, antiapoptotic effects of chemokine CCL2 have been demonstrated in NMDA and tat-protein (product of HIV-1) induced cell death in human mixed neuron-astrocytes cultures. The authors found that CCL2 reduced the extracellular level of glutamate and NMDA receptor 1 (NMDAR1) expression and pointed out the involvement of CCL2 in modulating the uptake, release, re‐synthesis, and metabolism of neurotransmitters through presynaptic chemokine receptors, and by regulating the expression of key neuronal proteins such as glutamate receptors [42]. It was also shown that noradrenaline increased CCL2 expression in astrocytes and that astrocyte-derived CCL2 was neuroprotective against NMDA or glutamate-induced excitotoxic damage and in the oxygen–glucose deprivation model [43]. Some studies suggested that CCL2 was upregulated upon ischemia, and directly acted on neurons leading to activation of pro-survival pathways via the transient receptor potential channels (TRPC), or via PI3K/Akt/NF-κB [44]. Other articles described the involvement of CCL2 in the prevention of methylmercury (MeHg)-induced cortical neuronal cell death. Blockade of the CCL2 using a specific antibody or antagonist of CCR2 induced an increase in necrotic and apoptotic neuronal cell death following MeHg incubation [45]. In general, results from the cell damage models demonstrate alternative regulatory roles of CCL2, other than inflammatory.

Based on the above-described multidirectional and important role of the CCL2-CCR2 axis in the brain, it can be expected that interference with this signaling pathway may disrupt numerous immune-neuronal processes that underlie the development of depressive disorders (Fig. 1).

## The role of the CCL2-CCR2 signaling in affective disorders—experimental and clinical studies

Sparse animal studies that describe the role of CCL2 in the pathophysiology of depression are based on stress procedures and their impact on the CNS, whereas research confirming the malfunction of CCL2 in depressed patients are investigating mainly the chemokine expression in the blood, plasma, or cerebrospinal fluid (CSF). Although the results of these examinations are to some extent contradictory, there are indications that disturbance of the homeostatic processes supervised by CCL2 may contribute to the onset of depressive symptoms.

The research carried out in our laboratory has proven that the prenatal stress procedure increases the levels of both the protein and mRNA for CCL2 and CCR2 in the hippocampus, as well as in the cortex of the tested rats [3]. Moreover, microglia isolated from 1–2-day-old prenatally stressed rats also demonstrated its adverse impact on the expression of the CCL2-CCR2 pair, which may suggest that harmful conditions during fetal life may result in disturbances of that axis appearing later in development [31]. Moreover, the prenatal stress procedure in mice caused an increase in the CCL2 synthesis in the placenta of treated dames and fetal brains, as well as decreased the levels of *Ccr2* mRNA in fetal brains. Interestingly, prenatally stressed germ-free mice displayed an increase in CCL2 synthesis in the placenta, but no changes in CCL2 protein levels and CCR2 expression were observed [46]. As reported by Wu et al. [47], the increased CCL2-CCR2 signaling in the nucleus accumbens (NAc) in mice plays a significant role in mediating the nociceptive behavior in neuropathic pain (induced by nerve injury) and associated depressive behavior. It was shown that social stress (repeated social defeat, RSD), established as a murine model of depression, increased CCL2 levels in the animal brain structures, such as the rostral cortex, hypothalamus, basal ganglia, and hippocampus via the mediation of monocytes trafficking from the bone marrow to the brain. Furthermore, in *Ccr2* knockout mice, the infiltration of macrophages to the brain was disrupted, resulting in the lack of anxiety-like behavior following RSD. [48].

Further evidence supporting this phenomenon came from the research showing that acute peripheral lipopolysaccharide (LPS) challenge in mice elicits the increased amounts of CCL2 mRNA and protein in the brain (hypothalamus, hippocampus), and upregulation of CCR2 expression by microglia and inflammatory monocytes. It entails the release of endogenous inflammatory factors in the brain and consequently causes the reduction of serotonin release by serotonergic neurons of the middle and dorsal raphe nucleus, which may be linked to the establishment of depressive phenotype [49]. Likewise, Le Thuc et al. [29] showed that intracerebral administration of LPS to mice increased the CCL2 synthesis and selective activation of CCR2 on MCH neurons. The change directly affected food intake and weight loss, which are key symptoms of sickness behavior. Those behavior-related characteristics were fully abolished in Ccr2 knockout mice or the CCR2 antagonist-treated animals. In contrast, in the model of depression conducted in rats, based on chronic variable stress, no changes in CCL2 levels in the hippocampus were observed [50]. Similarly, studies in chronic intermittent cold stress showed no effect of the stressful procedure on the expression of CCL2 in plasma, hypothalamus, and prefrontal cortex, but when the procedure was combined with the additional LPS stimulus, the chemokine levels were increased in the tested tissues [51].

Clinical data on disturbances in the CCL2-CCR2 signaling in patients with affective disorders provided contradictory information (Table 2). It seems that, in contrast to studies conducted in animal stress-based models of depression, these reports more often indicate decreased expression of the CCL2-CCR2 axis in peripheral tissues (less frequently in the brain as presented only in few post-mortem studies). Nevertheless, clinical trials in patients with an ongoing episode of major depression (MDD) (49 patients and 49 healthy volunteers matched for age and sex), showed elevated levels of CCL2 in the serum of the individuals with the disease [52]. Several studies indicate an increased level of CCL2, mainly in serum or plasma [53–56] or in the hippocampus [57] of patients with MDD, bipolar disorder (BD) [58], pregnant women suffering from depression accompanied by bacterial translocation [59] or with schizophrenia [60]. Furthermore, Goldsmith et al. [56] observed that elevated levels of CCL2 in the serum of patients with MDD correlated with the decreased psychomotor activity and worse results of neurocognitive tests. In patients with BD, the expression of this chemokine was associated with the cortical thickness in the right anterior cingulate cortex [61]. Other studies involving patients with this condition and their children who experienced symptoms of depression revealed that both groups were characterized by a higher CCL2 mRNA expression on isolated monocytes, which may indicate a role of this chemokine in the inheritance of this illness [62].

However, there are also data indicating the CCL2 decrease in the serum/plasma of patients with depressive disorders [63–65] or an unchanged level of this chemokine compared to healthy subjects in the serum of patients with BD [66, 67]. Interestingly, lower plasma levels of CCL2 in patients with MDD correlated with the occurrence of suicidal patients, while the level of CCL2 did not differ between patients with nonsuicidal MDD and healthy patients [63]. A similar feature, namely the decreased level of CCL2, was found in the CSF of suicide victims [68, 69].

It should not be forgotten that the serious course of coronavirus disease (COVID-19) caused by severe acute respiratory syndrome coronavirus 2 (SARS-CoV-2) is associated with cytokine storm, including elevated CCL2 levels, that further can activate neurons and microglia, and induce cognitive decline, mental stress or neuroinflammation [70].

Doubts regarding the level of CCL2 as a marker of depressive disorders are not dispelled by the meta-analyzes, which indicate higher levels of CCL2 in depressed patients compared to nondepressed individuals, but also indicate a lack of homogeneity of the examined studies [1, 71].

## CCL2-CCR2 axis in antidepressant therapy—experimental studies and clinical trials

In recent years, research not only has attempted to assess the role of chemokines in the pathophysiology of depressive disorders, but also has explored the possibility of regulating their levels through the use of pharmacotherapy. Relatively few studies examined the immunomodulatory effect of antidepressants on the levels of chemokines, however, the available reports suggest mostly their inhibitory effect on proinflammatory chemokines.

In the case of the CCL2 chemokine and CCR2, regarding the potential as a therapeutic target of antidepressant drugs, significant data have been provided by recent studies carried out in animal stress-based models of depression (Table 1). The authors showed that long-term antidepressant use not only alleviated behavioral dysfunction, but also weakened the function of IL-1β and IL-18 as well as the CCL2-CCR2 axis, upregulated by stress. Their results revealed that tianeptine (an atypical antidepressant) and venlafaxine (serotonin-norepinephrine reuptake inhibitor, SNRI) efficiently reduced the stress-induced increase in CCL2 levels in the hippocampus, while tianeptine further lowered stress-induced CCR2 levels in the hippocampus and cortex of rats [3]. Previously, this group found that tianeptine was effective in inhibiting CCL2 production by LPS-exposed primary microglial cells isolated from the cortices of 1–2-day-old Sprague–Dawley rat pups. A significant observation in the context of exerting an antidepressant effect through pathways related to CCL2 regulation in an animal model of depression was provided by Park et al. [72]. They showed that *Chamaecyparis obtusa* oil (EOCO) normalized, similarly to fluoxetine (selective serotonin reuptake inhibitor, SSRI), anxiety-like behavior induced by maternal separation (MS). The treatment was also associated with the inhibition of both the CCL2 gene and protein in the hippocampus of rats. Immunomodulatory properties of venlafaxine have been captured in a murine experimental autoimmune encephalomyelitis (EAE) model of multiple sclerosis. The authors proved that oral prophylactic and therapeutic administration of venlafaxine inhibited the clinical symptoms of the disease and, at the same time, decreased the level of CCL2 in peripheral tissues (peritoneal macrophages, splenocytes) and reduced the *Ccl2* gene expression in the spinal cord [73].

**Table 1:** CCL2 (MCP-1) protein and mRNA level after antidepressants administration in animal models of inflammation

Model	Result	Material	References
Noradrenaline (10 μM), 24 h	↑ MCP-1 protein concentration ↑ mRNA MCP-1 level ↓mRNA MCP-1 level	Rat cortical astrocytes Rat cortical microglia	[43]
Noradrenaline (10 μM) + LPS (0.1 μg/ml), 24 h	Partial normalization of changes in MCP-1 protein and mRNA level increased after LPS	Primary rat cortical astrocytes	[81]
Synthetic noradrenaline precursor L-DOPS (200 mg/kg daily/10 days) Desipramine (10 mg/kg), 5 h after injection	↑ MCP-1 protein concentration ↑ MCP-1 protein concentration	Mouse brain cortex Rat brain cortex	[27]
Desipramine (1–10 μM), 24 h Atomoxetine (40–50 μM), 24 h	↑ MCP-1 protein concentration ↑ mRNA MCP-1 level ↑ MCP-1 protein concentration ↑ mRNA MCP-1 level	Primary rat cortical astrocytes	[29]
Amitryptiline (16 mg/kg), a murine model of sepsis	↓ MCP-1 level	Serum samples	[74]
Tianeptine, Venlafaxine (10 mg/kg, daily)	Normalization the changes in MCP-1 level increased after prenatal stress	Hippocampus from adult rat offspring of stressed females	[3]
Amitryptiline (5 mg/kg), 7–14th post- sponge disks implantation days	↓ MCP-1 level	Polyether-polyurethane sponge disks implanted to mice	[75]
Reboxetine (10 mg/kg)/osmotic pumps/28 days in WT, 5xFAD and 5xFAD/CCL2KO mice	↑ mRNA MCP-1 level in WT mice after reboxetine treatment ↓mRNA IL-1β, MIP1α in 5xFAD/CCL2KO mice and after reboxetine administration	Brain cortex samples	[37]
Venlafaxine (60 mg/kg, daily/14 days), a murine model of experimental autoimmune encephalomyelitis	↓ mRNA MCP-1 level	Spinal cord tissue	[73]
Essential oil from *Chamaecyparis obtusa* (EOCO)—1–2 h inhalation/7 days, a rat model of maternal separation Fluoxetine (5 mg/kg), 7 days	Reduction of anxiety-related behavior similar to fluoxetine, normalization the changes in CCL2 mRNA and protein level increased after maternal separation	Hippocampal tissue	[72]

*LPS* lipopolysaccharide, *L-DOPS* L-threo-3,4-dihydroxyphenylserine, *WT* wild type, *5xFAD* Mouse Model of Alzheimer’s Disease, *CCL2KO* mice with deletion of CCL2, *IL-1β* interleukin 1β, *MIP1α* Macrophage Inflammatory Protein 1α, *EOCO* active components exert antigastropathic, anti-inflammatory, antioxidant activity and decrease the concentrations of stress hormones

The other studies that described the effectiveness of antidepressants on CCL2 inhibition covered the use of these drugs in different diseases other than depressive disorders. Indeed, amitriptyline (tricyclic antidepressants, TCAs) used in a murine sepsis model decreased the level of CCL2 and CXCL1 in the serum of the tested animals [74], also reduced the same chemokines in chronic inflammation caused by the implantation of biomaterials [75]. In addition, our research also indicates the effectiveness of antidepressants, such as fluoxetine, imipramine (TCAs) and desipramine (SNRIs) in inhibiting the secretion of the CCL2 chemokine, both by LPS- or TNF-α/IFN-γ-activated keratinocytes and dendritic cells used as a cellular model of contact hypersensitivity (in press).

Contrary to the above observations, researchers from the Madrigal group conducted a series of experiments showing the effects of noradrenaline (NA) and desipramine on the secretion and expression of CCL2 in both stress models and primary cultures isolated from the brain of animals [27, 29, 43]. They observed that acute administration of desipramine resulted in an increase of the concentration of CCL2 in the cortex of rats, which correlated with an elevation in NA levels in plasma [28]. A series of subsequent experiments also showed that the expression of both CCL2 mRNA and protein in primary astrocyte or microglia cultures was increased by drugs, such as desipramine, atomoxetine (both SNRIs), and NA itself [28, 30, 44]. Moreover, acute restrain stress did not cause a statistically significant increase in CCL2 in the cortex of the tested rats, but an elevated level of CCL2 was observed in stressed rats with metyrapone (a glucocorticoid inhibitor) co-administration. As the authors suggested, such a phenomenon can be explained by an increased discharge of glucocorticoids in response to stress. Indeed, these animals had elevated plasma corticosterone levels in response to stressful conditioning. It was confirmed in primary cultures of astrocytes, which corresponded to corticosterone treatment-induced reduction of CCL2 mRNA and protein expression [27]. Thus, the authors implicated that the therapeutic effect of SNRIs was due to the neuroprotective action of NA and was mediated, at least in part, by the induction and release of astrocyte-derived CCL2.

Importantly, clinical reports on the use of antidepressant drugs have not clarified the changes in CCL2 levels in patients with depressive disorder (Table 2). The meta-analysis performed by Kohler et al. [76] (study *N* = 5, subjects *N* = 163) showed that antidepressants might reduce peripheral levels of CCL2 patients with MDD. In the absence of unequivocal evidence for the importance of CCL2 as a biomarker of MDD, the results of recent research revealing a correlation between serum CCL2 levels and the therapeutic efficacy of the used antidepressants used are undoubtedly significant. Human studies presented that patients refractory to venlafaxine treatment showed significantly lower baseline CCL2 serum concentrations when compared to patients who became euthymic after treatment, although this level of CCL2 in the affected by MDD did not differ from the levels of this chemokine in healthy subjects [77].

**Table 2:** CCL-2 (MCP-1) chemokine—protein and mRNA profile in depressive patients/antidepressant treatment/healthy control—clinical studies

Objective	Participants	Diagnosis criteria	Material/samples	Results	References
Study associations of cytokines with depression and response to sertraline	23 patients with MDD, 25 controls	DSM-IV	Serum protein analysis	↑ CCL2 in MDD ↓ CCL2 after antidepressant treatment	[53]
Study associations of cytokines with MDD	49 patients with MDD, 49 controls	DSM-IV	Serum protein analysis	↑ CCL2 in depression	[52]
Study associations between serum chemokines and recurrent MDD in women with/without suicidal ideation	30 patients with MDD, 16 controls	DSM-IV	Serum protein analysis	↓ CCL2 in patients with suicidal ideation and MDD	[63]
The level of cytokines in patients with MDD compared with healthy subjects and their associations with antidepressant response	66 patients with MDD, 22 controls	DSM-IV	Serum protein analysis	↓ MCP-1 in depressive patients compared with healthy controls ↑ MCP-1 after antidepressant treatment	[65]
The level of chemokines in patients with MDD compared with healthy subjects	61 patients with MDD 61 controls	DSM-IV	Serum protein analysis	↓ MCP-1 in depressive patients compared with healthy controls	[64]
The level of chemokines in psychiatric patients in conjunction with a suicide attempt	137 patients with MDD 43 controls	DSM-III-R/DSM-IV	CSF protein analysis	↓ MCP-1 in suicide attempters compared with healthy controls	[69]
The level of chemokines in patients with MDD before and after eight-week treatment of fluoxetine hydrochloride in comparison with controls	34 patients with MDD 40 controls	DSM-IV	Serum protein analysis	↑ MCP-1 in depressive patients compared with healthy controls Normalization of the changes (↓ MCP1) after fluoxetine treatment	[46]
The level of chemokines in patients with treatment-resistant BDD received either escitalopram + celecoxib, or escitalopram + placebo	47 patients with BDD, 35 controls	HAMD-17	Plasma	MCP-1 levels were not altered in BDD patients ↓ MCP-1 in non-responders	[67]
The level of pro-inflammatory biomarkers in patients with MDD before and after eight-week treatment of venlafaxine in comparison with controls	22 patients with MDD, 14 patients with MDD treated with venlafaxine, 40 controls	DSM-IV/HAM-D	Serum protein analysis	↑ MCP-1 in MDD patients compared with healthy controls •No changes in MCP1 level after venlafaxine treatment	[54]
Meta-analysis of studies measured peripheral levels of cytokines and chemokines during antidepressant treatment in patients with MDD	Forty-five studies met inclusion criteria (*N* = 1517)		Serum samples	↓ MCP-1 after antidepressant treatment	[76]
Cytokine levels in healthy controls and depressed patients (responsive and refractory to treatment)	19 medicated patients with depression, 21 controls	ICD-10	Serum samples	No changes in MCP-1 level in MDD patients compared to healthy controls, ↓ MCP-1 in refractory	[77]
The single-cell analysis of microglia inflammation-associated molecules	6 medicated MDD cases as donors, five control donors without a history of depression	DSM-IV/III	Microglia isolated from human post-mortem brain tissue (four different regions)	No significant changes in the CCl2 gene expression, Enhanced homeostatic functions in medicated MDD cases	[80]
Cytokine and chemokine levels in depressed and medicated (escitalopram, 10 mg/day), 4 weeks) patients and healthy controls	12 patients with dysthymia, 12 with major depression, 20 healthy controls	DSM-IV	Serum samples	↑ MCP-1 in patients with dysthymic and major depression disorder No changes in MCP-1 level after escitalopram treatment	[79]
Cytokine and chemokine levels in depressed and medicated (escitalopram, 20–40 mg/day), 12 weeks) patients and healthy controls	19 patients with MDD, 27 healthy controls	DSM-IV	Plasma samples	↑ MCP-1 in MDD patients No significant changes in MCP-1 level after escitalopram treatment	[78]

*BDD* Bipolar Disorder Depression, *DSM-IV* The Diagnostic and Statistical Manual of Mental Disorders Fourth Edition, *HAMD* Hamilton Depression Scale, *ICD-10* the 10th revision of the International Classification of Diseases

It seems, therefore, that high levels of CCL2 are key to the efficient antidepressant effect of drugs, even though the suffering from depression are often characterized by elevated levels of this chemokine [53–56]. This hypothesis is supported by studies in which dysthymic as well as MDD patients treated with escitalopram [78, 79] or venlafaxine [54], despite initially elevated levels of CCL2 compared to healthy individuals, resulting in effective antidepressant response, but did not affect the CCL2 level. A similar observation was described in patients diagnosed with BD treated with the combination of escitalopram and celecoxib, where treatment responders had higher serum levels of CCL2 than those with more refractory depression [67]. The above reports may be supplemented by the results of post-mortem studies in patients treated with antidepressants, for whom the non-inflammatory microglia phenotype was suggested and these cells were characterized by increased expression of homeostatic markers [80].

## Conclusion

Both animal and human studies provide abundant evidence that the CCL2-CCR2 signaling plays a significant role in the development of depressive disorders. However, in the light of recent studies, the implication of CCL2-CCR2 signaling in the development and course of other affective disorders appears ambivalent. While mostly increased serum CCL2 level has been shown in patients suffering from depression, the lack of therapeutic effect of antidepressants was correlated with a low initial or final serum concentration of this chemokine. The recently appreciated non-inflammatory/homeostatic functions of the CCL2-CCR2 dyad create a rich new field for an understanding of the pathophysiological processes implicated in affective disorders. Assuming that, the CCL2-CCR2 axis action proves to play a significant role in the development of affective diseases, it may represent a new promising therapeutic target, however, further investigations are needed.
